# Unveiling the complexity of nanodiamond structures

**DOI:** 10.1073/pnas.2301981120

**Published:** 2023-05-30

**Authors:** Qi Zheng, Xian Shi, Jinyang Jiang, Haiyan Mao, Nicholas Montes, Nikolaos Kateris, Jeffrey A. Reimer, Hai Wang, Haimei Zheng

**Affiliations:** ^a^Materials Sciences Division, Lawrence Berkeley National Laboratory, Berkeley, CA 94720; ^b^School of Materials Science and Engineering, Southeast University 211189, Nanjing, P. R. China; ^c^Jiangsu Key Laboratory for Construction Materials, Southeast University 211189, Nanjing, P. R. China; ^d^Department of Mechanical Engineering, Stanford University, Stanford, CA 94305; ^e^Department of Chemical and Biomolecular Engineering, University of California, Berkeley, CA 94720; ^f^Department of Materials Science and Engineering, University of California, Berkeley, CA 94720

**Keywords:** nanodiamond, forbidden reflection, electron microscopy, atomic simulations, structural ordering

## Abstract

Understanding nanodiamond structures is of great scientific and practical interest. It has been a long-standing challenge to unravel the complexity underlying nanodiamond structures and to resolve the controversies surrounding their polymorphic forms. Here, we use transmission electron microscopy with high-resolution imaging, electron diffraction, multislice simulations, and other supplementary techniques to study the impacts of small sizes and defects on cubic diamond nanostructures. The experimental results show that common cubic diamond nanoparticles display the (200) forbidden reflections in their electron diffraction patterns, which makes them indistinguishable from new diamond (n-diamond). The multislice simulations demonstrate that cubic nanodiamonds smaller than 5 nm can present the *d*-spacing at 1.78 Å corresponding to the (200) forbidden reflections, and the relative intensity of these reflections increases as the particle size decreases. Our simulation results also reveal that defects, such as surface distortions, internal dislocations, and grain boundaries can also make the (200) forbidden reflections visible. These findings provide valuable insights into the diamond structural complexity at nanoscale, the impact of defects on nanodiamond structures, and the discovery of novel diamond structures.

## Significance

Uncovering the structural complexity of nanodiamonds has been a significant experimental and theoretical challenge. We show that cubic nanodiamonds of small sizes (e.g., <5 nm) display the characteristic (200) forbidden reflections in their electron diffraction patterns, which makes them indistinguishable from new diamond (n-diamond). Besides the size effect, our study demonstrates that various defects, including distortions, dislocations, and boundaries, can also make the (200) forbidden reflections visible. Thus, we supplement the explanation of n-diamond structure and reveal that using electron diffraction alone is insufficient to differentiate various diamond nanostructures. These findings shed light on the impact of order/disorder on nanodiamond structures at the single-atom level.

Diamond, the hardest natural material, has attracted great attention due to its scientific and industrial value (1, 2). Diamond is one of the most sought-after gemstones on earth. Naturally occurring diamonds, such as those originating deep in the earth’s mantle or meteoritic diamonds, are investigated in geological or extraterrestrial contexts (3). Diamonds can also be synthesized (4); synthetic diamonds have found a multitude of applications in mechanics (5), biomedicine (6), electronics (7), and photonics (8). Being an allotrope of carbon, diamond can exist in a wide variety of crystal structures that depend on the surrounding environment or processing techniques. Cubic diamond (c-diamond) is the most common diamond crystal structure (9). It comprises a tetrahedrally connected *sp*^3^-bonded network of carbon atoms that form six-membered rings in the “chair” conformation, as in the cyclohexane molecule, and are in turn linked with covalent bonds. Besides c-diamond, various other structures have been reported, including new diamond (n-diamond) (10), hexagonal diamond (h-diamond or lonsdaleite) (11), and i-carbon (10) (*SI Appendix*, Fig. S1). In all diamond forms, regions with *sp*^3^- and *sp*^2^-structured units are coherently bonded together through covalent linkage (12).

Extensive studies have demonstrated the structural complexity of nanodiamonds. In particular, the n-diamond was reported as a new diamond-like structure (10), and it has been used as a marker of asteroidal impacts. One of the interests in n-diamond stems from the fact that n-diamond nanoparticles have been identified in multiple samples of the Younger–Dryas (YD) boundary sediment layer, which supports the hypothesis that one or more cometary airbursts barraged North America (13–15), causing a catastrophic climate shift. However, the existence and the origin of n-diamond have been contested in several studies (16–18). Similarly, a recent study on lonsdaleite diamond, which was also found in the YD boundary sediment (14), suggested that the claim of its hexagonal crystal symmetry can be faulty and the identified lonsdaleite diamond may instead be twinned cubic diamond (19).

Nanodiamond structures are typically characterized using X-ray diffraction (XRD), Raman spectroscopy (20, 21), or transmission electron microscopy (TEM) techniques (22). In bulk c-diamond, some reflections, such as the (200) reflections at the *d*-spacing of 1.78 Å, are forbidden and thus invisible in the electron diffraction patterns (10). The appearance of such forbidden spots (*h* + *k + l = 4n + 2*) in n-diamond has been considered a key feature that differentiates it from c-diamond, despite the fact that the forbidden reflections can also appear in thick c-diamond samples (*SI Appendix*, Fig. S2) (23) due to double diffraction (*SI Appendix*, Fig. S3) (24). Therefore, the approach of using diffraction features to determine diamond structures needs to be re-evaluated.

In this work, we provide a systematic study of the size and defect impact on c-diamond nanostructure by examining the diffraction features using aberration-corrected high-resolution TEM, molecular dynamics, and multislice simulations. We show that structural complexities arise from small sizes, surface distortions, and structural imperfections in c-diamond nanoparticles, resulting in the appearance of the (200) forbidden reflections in the diffraction patterns of cubic nanodiamonds smaller than 5 nm. Our results provide new insights into the identification of diamond structures by highlighting the structural heterogeneity in nanodiamonds, which may bring impact on the interpretation of n-diamond as a marker of asteroidal impacts.

## Results and Discussions

**Fig. 1.**
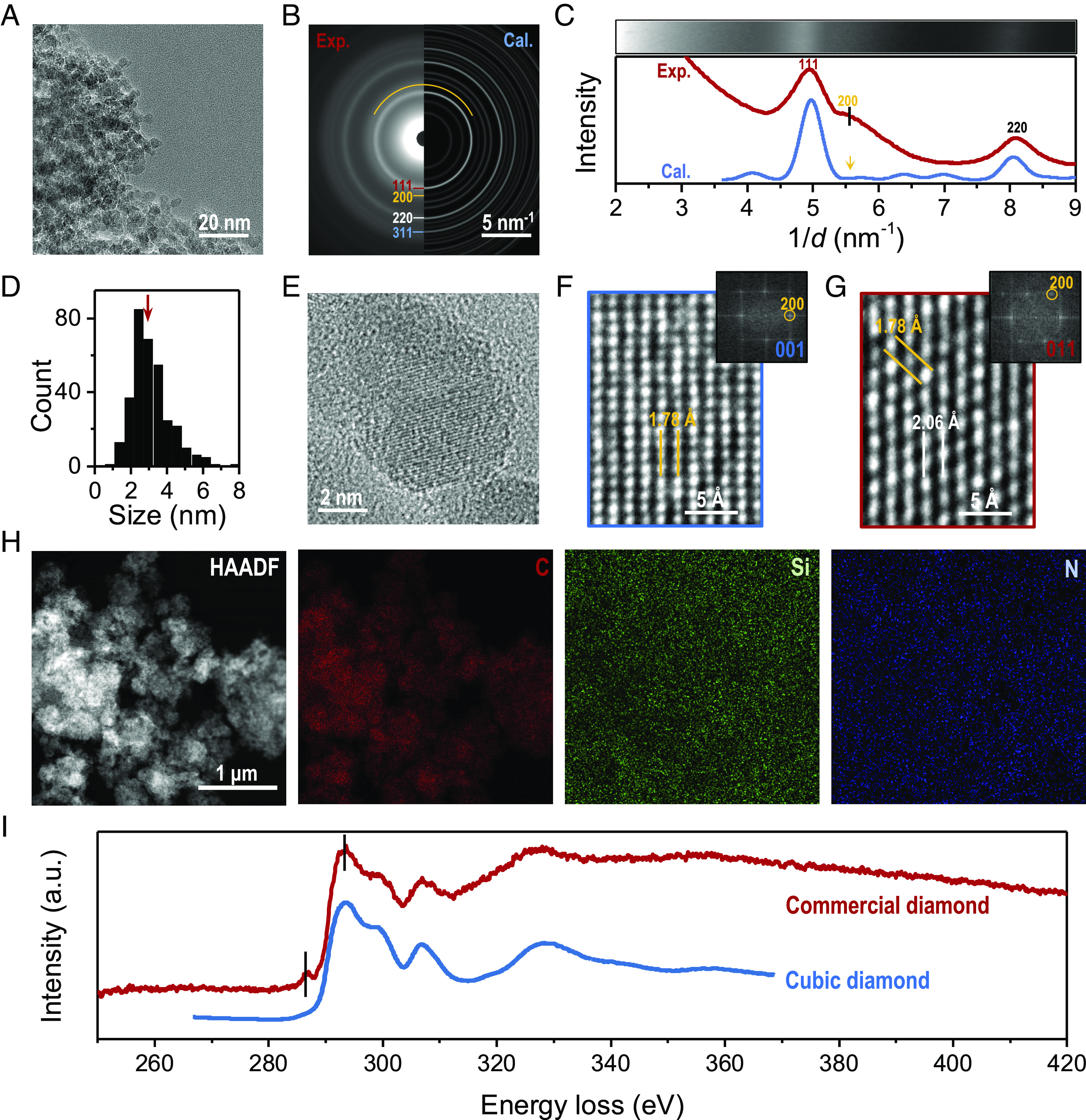
Morphology and elemental analysis of commercial nanodiamond particles. (*A*) A low-magnification TEM image of nanodiamond agglomerates. (*B*) The corresponding selected area electron diffraction pattern, with different lattice planes highlighted. The *Left* panel is from the experiment and the *Right* is calculated based on c-diamond polycrystals. (*C*) Electron diffraction pattern visualized as a function of azimuthal angle and its radial intensity profiles derived from *B*. (*D*) The particle size distribution of nanodiamonds in *A*. The particles are traced based on image segmentation analysis. (*E*) A TEM image of an individual c-diamond nanoparticle. (*F* and G) High-resolution TEM images of nanodiamond domains from the [001] and [011] zone axes, respectively. The *Insets* in *F* and *G* are the corresponding FFT patterns with visible (200) spots. The *d*-spacings at 1.78 and 2.06 Å are labeled as (200) and (011) lattice planes, respectively. (*H*) Energy-dispersive X-ray spectroscopy (EDS) maps of nanodiamond particles. A high-angle annular dark field image of nanodiamond particles, and the related elemental mappings of carbon (C), silicon (Si), and nitrogen (N) elements. The nanodiamond sample is dropped onto a SiN_x_ membrane to exclude potential carbon background often observed when using traditional carbon-film TEM grids. (*I*) Electron energy loss spectroscopy (EELS) core-loss spectra of the nanodiamond sample. The cubic-diamond reference spectrum (https://eelsdb.eu/spectra/diamond/) is stacked for peak identification. The peak at 292 eV represents the 1 s to σ* transition of C-C bonds. An additional peak at 287 eV is observed from the nanodiamond sample, which is attributed to the 1 s to σ* (C-H) or *sp*^2^-bonded C. The EELS intensity was normalized in arbitrary unit.

We investigate cubic nanodiamonds of small sizes (< 5 nm roughly in diameter) by high-resolution imaging using an aberration-corrected transmission electron microscope (ThemIS). Commercially available cubic nanodiamonds, which were produced from a multicathode direct current plasma-assisted chemical vapor deposition process at a temperature of 3,000 K and a pressure of 100 Torr (25, 26), were used as acquired. Our atomic-resolution imaging and multimodal analytical characterization are illustrated in Fig. 1. In Fig. 1*A*, the nanodiamond particles are aggregated due to electrostatic interactions (27). They are free of surface graphite or residual soot that may appear in some diamond nanoparticles (28). The Raman spectrum exhibits a notable diamond (D) peak and an asymmetric G band, with no evidence of a 2D or D′ band (*SI Appendix*, Fig. S4) that would be indicative of graphitic structures. Notably, a diffusive (200) diffraction ring at 0.56 Å^–1^ (i.e., 1.78 Å) is observed in the diffraction pattern (Fig. 1*B*), which is expected to be absent in ordinary c-diamond (space group: Fd-3m). We confirm that the experimental diffraction pattern is consistent with the calculated pattern of c-diamond polycrystals at a size of 3 nm. A distinct (200) peak can also be noticed in the derived radial intensity profile in Fig. 1*C*. The nanodiamond particles imaged have an average diameter of 3.5 nm (Fig. 1*D*), smaller than those studied previously (29–31). At this size, double diffraction is unlikely to occur (23). We later focused on the individual nanodiamond particle (Fig. 1*E*) instead of the ensemble average. The *d*-spacing of the (200) lattice plane at 1.78 Å can be measured in the high-resolution TEM images (Fig. 1 *F* and *G*). In detail, a cubic lattice can be discerned from the [001] zone axis (Fig. 1*F*); a hexagonal lattice is revealed at the [011] zone axis (Fig. 1*G*). The (200) forbidden spots are observed also from the corresponding fast Fourier transform (FFT) patterns. Hence, the diamond nanoparticles imaged are decidedly c-diamond, yet with the (200) forbidden reflections.

We also performed energy-dispersive X-ray spectroscopy (EDS) mapping using a PELCO® silicon nitride (SiN_x_) membrane substrate of 8 nm thickness. As shown in Fig. 1*H*, the nanodiamond samples are free of impurities (see *SI Appendix*, Fig. S5 for the full EDS spectrum). The weak signals for N and Si arise from the SiN_x_ membrane. The structure of cubic nanodiamond is also confirmed through EELS, showing good agreement with the reference spectrum of c-diamond (Fig. 1*I*) (32). The small σ* peak at 287 eV is associated with the 1s to σ* (C-H) transition with a small fraction of *sp^2^*-bonded carbon atoms (33), while the relatively broad σ* peak at 292 eV originates from C-C bonds through a 1 s to σ* transition (34, 35).

**Fig. 2.**
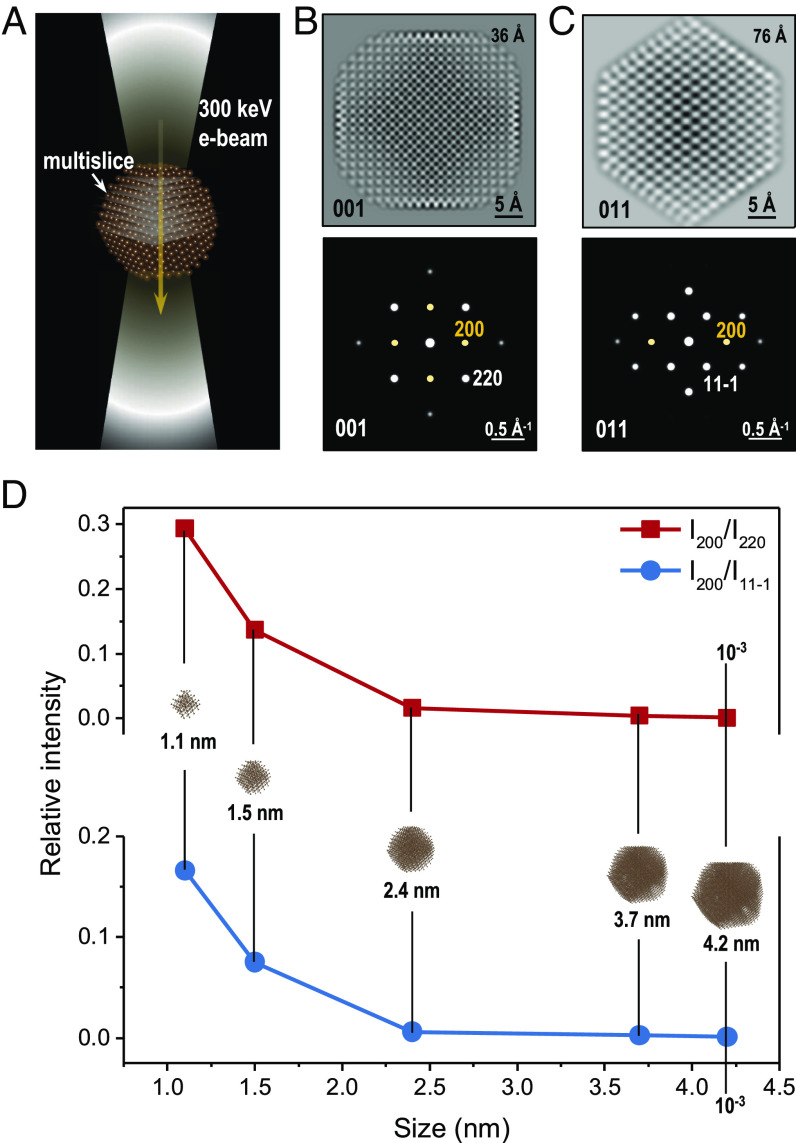
Smaller nanodiamond particle enhances the intensity of (200) spots. (*A*) An illustration of multislice simulation setup. Simulated TEM images and diffraction patterns of a 3.7 nm diamond particle from (*B*) the [001] and (*C*) the [011] zone axes. The defocus values of the simulated TEM images are labeled on the top, at 36 and 76 Å, respectively. The (200) spots are highlighted, which can be observed from both the [001] and [011] zone axes. Also, the adjacent (220) and (11-1) are marked as reference spots to calculate the relative intensity of the forbidden reflections. (*D*) The relative intensity of the (200) spot as a function of nanodiamond particle size. The *Insets* are the constructed atomic structures of nanodiamonds from 1 nm to ~5 nm. Nanodiamonds >5 nm in size exhibit the relative intensity <10^–3^, which might not be detected in the diffraction experiments.

To validate our experimental results, we carried out a series of multislice simulations. As illustrated in Fig. 2*A*, a nanodiamond particle is constructed in accordance with the cubic diamond model (36) and multislice calculations are performed to achieve the corresponding TEM images in the real space (37), and the diffraction patterns in the reciprocal space (38, 39). The simulations were performed at 300 keV and an optimized slice separation of 0.2 nm was selected (*SI Appendix*, Fig. S6). All parameters employed in the simulations were consistent with the ThemIS microscope used in the experiments. Fig. 2 *B* and *C* present the results of a 3.7 nm nanodiamond particle comprising of 4,820 carbon atoms. Importantly, the (200) forbidden spots can be detected at both the [001] and the [011] zone axes. As the overall spot intensity may vary with the size of diamond nanoparticles, we evaluated the intensity of the (200) spot by normalizing it against those of the adjacent spots, for example, *I*_200_/*I*_220_ for the [001] zone axis and *I*_200_/*I*_11-1_ for the [011] zone axis. We found that the relative intensity of the (200) spot increases as the size of the cubic nanodiamond becomes smaller at both the [001] and the [011] zone axes (Fig. 2*D*).

We further conducted calculations on a variety of c-diamond nanoparticles over a range of geometries. The results demonstrate that it is a common phenomenon for (200) spots to be visible for c-diamond nanoparticles smaller than 5 nm. In all cases, the relative intensity of the (200) spots increases as particle size decreases (*SI Appendix*, Fig. S7). Additional benchmark simulations also corroborate our calculation results. *SI Appendix*, Fig. S8 demonstrates the relative intensity of the (200) spot versus the thickness of a c-diamond nanosheet, where a brighter forbidden spot is found in a thicker sample, consistent with the double-diffraction effect. Therefore, our simulations include the double diffraction effect, as well as additional effects [e.g., extinction (40)]. As double diffraction contributes to the relative intensity of (200) spots more significantly in larger diamond particles, our discovery of the appearance of (200) spots in small nanodiamonds and their increased relative intensity suggests that the (200) spots are otherwise not always forbidden for cubic diamonds at all sizes. They are in fact omnipresent in smaller c-diamond nanoparticles (<5 nm) at a much lower intensity.

**Fig. 3.**
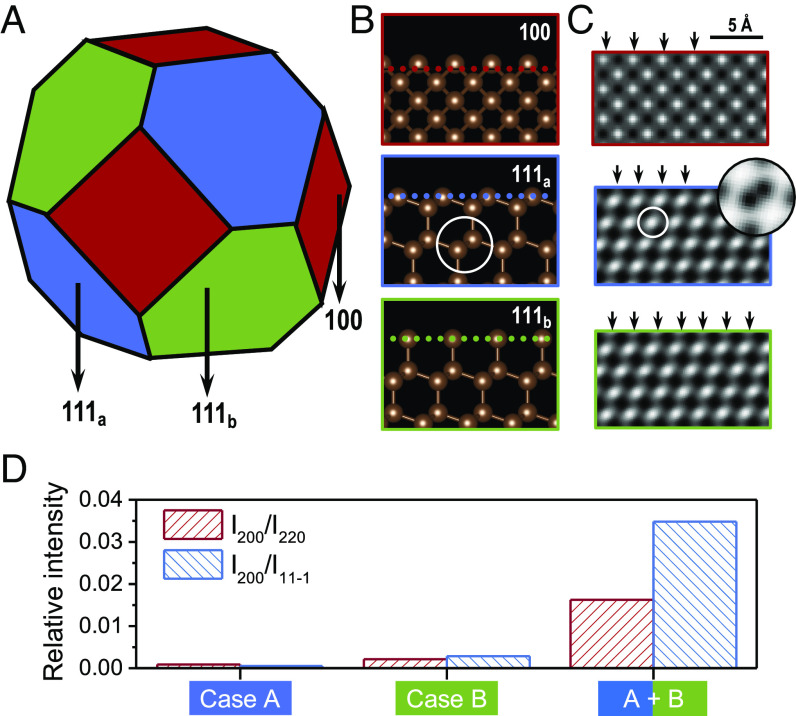
Surface asymmetry induces stronger (200) spots. (*A*) An illustration of a 3.7 nm truncated octahedral nanodiamond particle. The particle is composed of {100} and {111} facets, while the {111} facets can be either terminated by sixfold rings ({111_a_}) or dangling carbon atoms ({111_b_}). (*B*) Atomic structures of the cleaved {100} and {111} facets with (*C*) the corresponding TEM images. The terminated atoms are highlighted by arrows. Each bright dot represents an individual atom column. (*D*) The relation between different surface combinations and the relative intensity of the (200) spot. Here, *Case A* represents the particle with all {111} facets treated as {111_a_}; *Case B* considers the {111} facets as {111_b_}. *A + B* indicates an asymmetrical case that is consistent with the geometry shown in *A*: half of the {111} facets are {111_a_} and the rest are {111_b_}. The {100} facets are all fixed by the termination option shown in *B*. The {111} facets of nanodiamonds in Fig. 2 are all cleaved as in *Case A*.

Nanoparticles have a large surface area-to-volume ratios; the atomic arrangement on the particle surfaces is often defective (41), e.g., with the presence of dangling bonds, surface steps, atomic displacements, surface strain, and incomplete surface layers (such as wedges, steps). Surface termination mismatch can often result in the appearance of kinematically forbidden reflections (42). Here, we examine surface effects on the (200) forbidden reflections. Fig. 3 illustrates surface effects for a 3.7 nm c-diamond nanoparticle. As a classical geometry, a truncated octahedral diamond particle is constructed (Fig. 3*A*), consisting of {100} and {111} facets (43). Two options exist for cleaving the {111} facet, either exposing entire six-fold rings ({111_a_}) or forming dangling carbon bonds ({111_b_}), in contrast to the sole cleavage of the {100} facet terminated by the cubic lattice (Fig. 3*B*). The above choices exist in the experiments with the corresponding atomic TEM images simulated shown in Fig. 3*C*, which illustrates the terminated surfaces, respectively (marked by the arrows). Note that the bright dot in the {100} facet represents a single atom column, while those in the {111} facet indicate diatomic columns with an overlap at a –44 Å defocus value (the contrast of the inset is inverse to better identify the diatoms). As shown in Fig. 3*D*, we demonstrate that the relative intensity of the (200) spot is significantly enhanced, by over one order of magnitude, in the case of an asymmetrical surface, half of which is composed of a {111_a_} facet and the other half of a {111_b_} facet. Meanwhile, surface roughness influences the presence of forbidden spots. A smoother surface dominated by the hexagonal carbon rings ({111_a_}) can dim the (200) spot. In contrast, the dangling carbon bonds ({111_b_}) brighten the (200) spot. Such effects are exceptionally amplified for nanodiamond particles smaller than 5 nm.

**Fig. 4.**
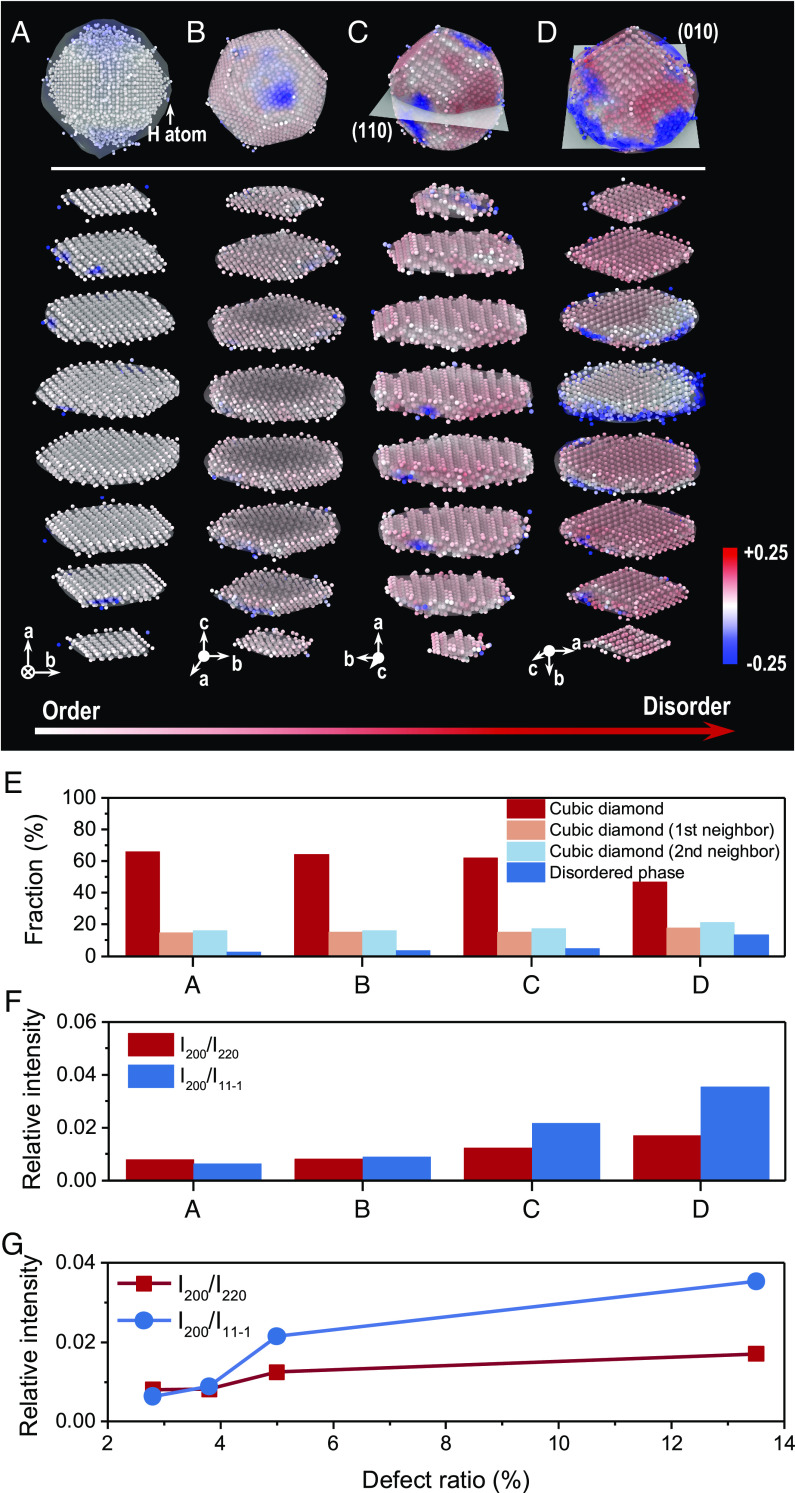
Structural defects of nanodiamonds enhance the relative intensity of (200) spots. (*A*–*D*) Atomic structures of nanodiamond particles. All nanoparticles are optimized and relaxed in a 5 ns NVE run after cleaving facets and introducing defects. (*A*) Hydrogen-capped nanodiamond with dangling carbon atoms on the surface stabilized by hydrogen atoms. The H atoms are visualized as an isosurface. (*B*) Relaxed nanodiamond that undergoes a 5 ns geometry relaxation [i.e., the same 3.7 nm nanodiamond in Figs. 2 and 3 (in *Case A*) without relaxation]. Surface atoms are more disordered and display higher strains. (*C*) Twinned nanodiamond. The top and bottom parts are slightly offset along the (110) plane within the structure. (*D*) Misoriented nanodiamond. The orientations of the top and bottom domains are different along the (010) plane, causing the grain interface in the middle to be highly strained. All the nanodiamonds are 3.7 ± 0.1 nm in diameter. The *Top* panels show a 3D view of the nanodiamonds, followed by the sliced diamond structures listed. Atoms are colored by volumetric strain from −0.25 to 0.25. Note that a tendency from ordered to disordered structures can be distinguished based on atomic strain distribution among (*A*–*D*). (*E*) Quantitative analysis of the atomic fraction of diamond phases including the ordered cubic lattice atoms and the disordered phase. (*F*) The relative intensity of the (200) spot versus different nanodiamond particles in *A*–*D*. (*G*) The relative intensity of the (200) spot as a function of the defect ratio of diamond structures. The defect ratio is calculated based on the relative proportion of the disordered phase.

Apart from the surface effect, structural defects can also enhance the relative intensity of (200) spots. We have further constructed a set of c-diamond nanoparticles with defects varying from surface ligand modification, to twinning, dislocation, and grain misorientation, as shown in Fig. 4 *A*–*D*. Different from the pristine nanodiamond particles mentioned above, we performed ReaxFF molecular dynamics (MD) simulations on these defected nanodiamonds (~3.7 nm) in a microcanonical ensemble, to relax the internal stress and optimize each of the structures (*SI Appendix*, Fig. S9). These nanodiamonds may be regarded as more realistic models concerning the commonly found features in experiments (*SI Appendix*, Fig. S10 lists the experimental results). For example, the dangling carbon atoms on the surface are commonly stabilized by ligands. From our EELS spectrum (Fig. 1*I*), the diamond nanoparticles contain some C-H bonds (i.e., the minor peak at 287 eV), which is indicative of surface modification. FTIR spectra of the same sample (*SI Appendix*, Fig. S11) further indicate that the surfaces of the nanoparticles are terminated by hydrogen with significant alkyl groups. Here, the hydrogen-capped diamond displays a better crystalline structure (Fig. 4*A*) with negligible surface strains, in comparison with that of a surface with the dangling C atoms only. Fig. 4*B* refers to the relaxed nanodiamond after a sufficient MD relaxation time (5 ns). Atoms on the surface are more disordered, as commonly seen in metal nanoparticles such as platinum and gold (44, 45). A twin is artificially introduced through lattice gliding along the (110) plane (Fig. 4*C*). Such twinning takes the form of “translation gliding” (46), whereby one or more rows of atoms are displaced laterally along the glide plane, taking up a small displacement by each row within the lattice (*SI Appendix*, Fig. S12). We also created a grain boundary inside the nanodiamond in Fig. 4*D*. The orientations of the top and bottom domains are different along the (010) plane (*SI Appendix*, Fig. S13), leading to strain being highly concentrated at the grain boundary with lattice misorientations (47). These nanodiamond structures are rendered by the volumetric strain, and thus we have an overview that the structure becomes more disordered when more defects are introduced.

The disorder-order fraction is quantified based on the coordination method (*SI Appendix*, Fig. S14 and Table S1). It is not surprising to see that the fraction of the disordered phase increases when more defects are introduced in the nanodiamond structures (Fig. 4*E*). The corresponding diffraction simulations in Fig. 4*F* suggest that structural defects can markedly enhance the relative intensity of (200) spots. The (200) intensity of the crystalline H-capped diamond is only 0.01, while it increases to 0.04 when a grain misorientation defect is presented. Notably, a direct relation between the (200) intensity and the defect ratio (the proportion of the disordered phase) can be obtained, as shown in Fig. 4*G*. Note that similar results can be observed frequently for the kinematically forbidden (200) spots appearing in silicon crystal with dislocations (48, 49). Considering that the (200) spot can be clearly distinguished in the c-diamond nanoparticles (Fig. 1), we further carried out NMR measurements on the experimental sample. The results (*SI Appendix*, Fig. S15) confirm the presence of defects inside the nanodiamonds with different bonding preferences (50, 51). Based on the results, we conclude that the (200) forbidden spots can also be induced by structural defects, and the relative intensity of the (200) spots can increase with increasing levels of defects in c-diamond nanoparticle structures.

In summary, we have uncovered various possible origins of the (200) forbidden spots in cubic nanodiamonds through a systematic study with multislice simulations, high-resolution TEM imaging, electron diffraction, and other supplementary experiments. Our findings show that c-diamond nanoparticles smaller than 5 nm can retain the forbidden reflections, and the relative intensity of the forbidden reflections increases as the size of nanoparticle decreases with or without defects. Furthermore, the relative intensity of the forbidden reflections is expected to be amplified with an increase in surface and structural defects. In particular, the observed forbidden reflections in cubic nanodiamonds can result from the dynamical diffraction processes due to crystal asymmetries, defects, grain boundaries, and other structural complexities. The findings may have broader applicability to other crystals with similar crystal symmetries. Our work conclusively shows cubic nanodiamonds display electron diffraction patterns indistinguishable from n-diamond, and hence, it has important implications for the identification of nanodiamond structures in broad applications.

## Materials and Methods

Cubic diamond nanoparticles (C, >98%, 3 nm) were purchased from Sigma Aldrich Chemical Reagent Co., Ltd and used as received. TEM grids were purchased from Electron Microscopy Sciences, Ted Pella, Inc. We acquired the TEM images using a ThemIS transmission electron microscope with a Thermo Fisher Scientific Ceta CMOS camera, at an electron dose rate of 325 e^−^Å^2^s^−1^, to avoid beam damage-induced structural change (*SI Appendix*, Fig. S16). The microscope was operated at 300 keV with the Bruker SuperX EDS detector, allowing rapid chemical identification. The diffraction patterns were collected in a three-condenser TEM mode at a parallel beam illumination condition. A 10-μm selected area aperture and an exposure time of 200 ms at a spot size of 6 was applied throughout the electron diffraction experiment. The camera length was selected at 770 mm with a 0.00 mrad convergence angle. We further performed “Sum Calculation” on diffraction patterns to highlight the (200) ring. In detail, 10 diffraction patterns with same experimental conditions were aligned and summed using the absolute intensity values, in the Fiji package. EELS analysis was performed on FEI Tecnai F20 UT at 200 kV in STEM mode with 0.15 eV energy resolution. The energy dispersion was set to 0.02 eV per channel for the near-edge structure of the C K-edge.

Diffraction patterns of c-diamond polycrystals were calculated using CrystalMaker 10.8. We performed the simulations at 300 keV, at the camera length of 770 mm and a 0.00 mrad convergence angle. The spot size of the detector was 0.02 Å^–1^ with the saturation at 100 and a Gamma value of 2. The diamond sample thickness was set at 3 nm. Some atom distortions and a 2% isotropic strain were introduced into the sample to reflect more realistic c-diamond particles.

The multislice simulations were performed at 300 keV. The chromatic aberration coefficient was set at *C_c_* = 1.4 mm, the spherical aberration coefficient used was *C_s_* = 1 mm. The energy spread for the microscope was set at 1.6 eV with an aperture diameter of 10.94 nm^–1^. The aperture was centered at the reciprocal space origin. The slice separation was 0.2 nm for all multislice calculations. Based on various slice separation distances, we estimated the relative error of our simulation to be approximately 8%.

For molecular dynamics simulations, the initial c-diamond crystal structure was obtained from Bindzus’s measurements (36). Molecular dynamics simulations were performed on the LAMMPS platform (52), using a velocity Verlet algorithm with a time step of 0.1 fs, with temperature fluctuating around 300 K. The ReaxFF force field was used for potential energy evaluation (53). A microcanonical ensemble was employed for 5 ns, sufficient to relax the structure. All the atomic configurations were visualized in OVITO (54). The diamond nanoparticles in Fig. 4 are relaxed; those in Figs. 2 and 3 are pristine nanodiamonds without structural relaxation.

## Supplementary Material

Appendix 01 (PDF)

## Data Availability

All study data are included in the article and/or *SI Appendix*.

## References

[1] Q. Huang , Nanotwinned diamond with unprecedented hardness and stability. Nature **510**, 250–253 (2014). 24919919 10.1038/nature13381

[2] Y. Yue , Hierarchically structured diamond composite with exceptional toughness. Nature **582**, 370–374 (2020). 32555490 10.1038/s41586-020-2361-2

[3] F. Nestola , Impact shock origin of diamonds in ureilite meteorites. Proc. Natl. Acad. Sci. U.S.A. **117**, 25310–25318 (2020). 32989146 10.1073/pnas.1919067117 PMC7568235

[4] L. Basso, M. Cazzanelli, M. Orlandi, A. Miotello, Nanodiamonds: Synthesis and application in sensing, catalysis, and the possible connection with some processes occurring in space. Appl. Sci. **10**, 4094 (2020).

[5] C. Dang , Achieving large uniform tensile elasticity in microfabricated diamond. Science **371**, 76–78 (2021). 33384375 10.1126/science.abc4174

[6] B. S. Miller , Spin-enhanced nanodiamond biosensing for ultrasensitive diagnostics. Nature **587**, 588–593 (2020). 33239800 10.1038/s41586-020-2917-1

[7] Z. Shi , Metallization of diamond. Proc. Natl. Acad. Sci. U.S.A. **117**, 24634–24639 (2020). 33020306 10.1073/pnas.2013565117 PMC7547227

[8] I. Aharonovich, A. D. Greentree, S. Prawer, Diamond photonics. Nat. Photon. **5**, 397–405 (2011).

[9] A. Banerjee , Ultralarge elastic deformation of nanoscale diamond. Science **360**, 300–302 (2018). 29674589 10.1126/science.aar4165

[10] H. Hirai, K. Kondo, Modified phases of diamond formed under shock compression and rapid quenching. Science **253**, 772–774 (1991). 17835494 10.1126/science.253.5021.772

[11] C. Frondel, U. B. Marvin, Lonsdaleite, a hexagonal polymorph of diamond. Nature **214**, 587–589 (1967).

[12] K. Luo , Coherent interfaces govern direct transformation from graphite to diamond. Nature **607**, 486–491 (2022). 35794481 10.1038/s41586-022-04863-2 PMC9300464

[13] D. J. Kennett , Nanodiamonds in the Younger Dryas boundary sediment layer. Science **323**, 94 (2009). 19119227 10.1126/science.1162819

[14] D. J. Kennett , Shock-synthesized hexagonal diamonds in Younger Dryas boundary sediments. Proc. Natl. Acad. Sci. U.S.A. **106**, 12623–12628 (2009). 19620728 10.1073/pnas.0906374106 PMC2722287

[15] I. Israde-Alcántara , Evidence from central Mexico supporting the Younger Dryas extraterrestrial impact hypothesis. Proc. Natl. Acad. Sci. U.S.A. **109**, E738–E747 (2012). 22392980 10.1073/pnas.1110614109 PMC3324006

[16] P. Németh, L. A. J. Garvie, P. R. Buseck, Twinning of cubic diamond explains reported nanodiamond polymorphs. Sci. Rep. **5**, 1–8 (2015). 10.1038/srep18381 PMC4680963 26671288

[17] T. L. Daulton, N. Pinter, A. C. Scott, No evidence of nanodiamonds in Younger-Dryas sediments to support an impact event. Proc. Natl. Acad. Sci. U.S.A. **107**, 16043–16047 (2010). 20805511 10.1073/pnas.1003904107 PMC2941276

[18] H. Tian, D. Schryvers, P. Claeys, Nanodiamonds do not provide unique evidence for a Younger Dryas impact. Proc. Natl. Acad. Sci. **108**, 40–44 (2011). 21173270 10.1073/pnas.1007695108 PMC3017148

[19] P. Németh , Lonsdaleite is faulted and twinned cubic diamond and does not exist as a discrete material. Nat. Commun. **5**, 1–5 (2014). 10.1038/ncomms6447 25410324

[20] V. N. Mochalin, O. Shenderova, D. Ho, Y. Gogotsi, The properties and applications of nanodiamonds. Nat. Nanotechnol. **7**, 11–23 (2012). 10.1038/nnano.2011.209 22179567

[21] I. Pope , Coherent anti-Stokes Raman scattering microscopy of single nanodiamonds. Nat. Nanotechnol. **9**, 940–946 (2014). 25305746 10.1038/nnano.2014.210 PMC4990125

[22] P. Németh , Complex nanostructures in diamond. Nat. Mater. **19**, 1126–1131 (2020). 32778814 10.1038/s41563-020-0759-8

[23] A. Nie , Approaching diamond’s theoretical elasticity and strength limits. Nat. Commun. **10**, 1–7 (2019). 31797924 10.1038/s41467-019-13378-w PMC6892892

[24] L. Reimer, Transmission Electron Microscopy: Physics of Image Formation and Microanalysis (Springer, 2013).

[25] W.-S. Lee, K.-W. Chae, K. Y. Eun, Y.-J. Baik, Generation of pulsed direct-current plasma above 100 torr for large area diamond deposition. Diam. Relat. Mater. **10**, 2220–2224 (2001).

[26] J.-K. Lee , The large area deposition of diamond by the multi-cathode direct current plasma assisted chemical vapor deposition (DC PACVD) method. Diam. Relat. Mater. **11**, 463–466 (2002).

[27] A. S. Barnard, E. Ōsawa, The impact of structural polydispersivity on the surface electrostatic potential of nanodiamond. Nanoscale **6**, 1188–1194 (2014). 24302124 10.1039/c3nr05344j

[28] A. Krueger, Diamond nanoparticles: Jewels for chemistry and physics. Adv. Mater. **20**, 2445–2449 (2008).

[29] F. Banhart, P. M. Ajayan, Carbon onions as nanoscopic pressure cells for diamond formation. Nature **382**, 433–435 (1996).

[30] J. Xiao, G. Ouyang, P. Liu, C. X. Wang, G. W. Yang, Reversible nanodiamond-carbon onion phase transformations. Nano Lett. **14**, 3645–3652 (2014). 24823241 10.1021/nl5014234

[31] Y.-K. Tzeng , Vertical-substrate MPCVD epitaxial nanodiamond growth. Nano Lett. **17**, 1489–1495 (2017). 28182433 10.1021/acs.nanolett.6b04543

[32] A. Gloter, A. Douiri, M. Tence, C. Colliex, Improving energy resolution of EELS spectra: An alternative to the monochromator solution. Ultramicroscopy **96**, 385–400 (2003). 12871803 10.1016/S0304-3991(03)00103-7

[33] L. Ponsonnet , EELS analysis of hydrogenated diamond-like carbon films. Thin Solid Films **319**, 97–100 (1998).

[34] S. D. Berger, D. R. McKenzie, P. J. Martin, EELS analysis of vacuum arc-deposited diamond-like films. Philos. Mag. Lett. **57**, 285–290 (1988).

[35] F. Langenhorst, V. L. Solozhenko, ATEM-EELS study of new diamond-like phases in the B-C–N system. Phys. Chem. Chem. Phys. **4**, 5183–5188 (2002).

[36] N. Bindzus , Experimental determination of core electron deformation in diamond. Acta Crystallogr. Sect. A **70**, 39–48 (2014). 10.1107/S2053273313026600 24419169

[37] A. Gómez-Rodríguez, L. M. Beltrán-del-Río, R. Herrera-Becerra, SimulaTEM: Multislice simulations for general objects. Ultramicroscopy **110**, 95–104 (2010). 19853997 10.1016/j.ultramic.2009.09.010

[38] A. Chuvilin, U. Kaiser, On the peculiarities of CBED pattern formation revealed by multislice simulation. Ultramicroscopy **104**, 73–82 (2005). 15935917 10.1016/j.ultramic.2005.03.003

[39] J. Reyes-Gasga, A. Gómez-Rodríguez, X. Gao, M. José-Yacamán, On the interpretation of the forbidden spots observed in the electron diffraction patterns of flat Au triangular nanoparticles. Ultramicroscopy **108**, 929–936 (2008). 18501517 10.1016/j.ultramic.2008.03.005

[40] J. Gjønnes, A. F. Moodie, Extinction conditions in the dynamic theory of electron diffraction. Acta Crystallogr. **19**, 65–67 (1965).

[41] L. Wang , Tunable intrinsic strain in two-dimensional transition metal electrocatalysts. Science **363**, 870–874 (2019). 30792302 10.1126/science.aat8051

[42] D. P. Woodruff, Solved and unsolved problems in surface structure determination. Surf. Sci. **500**, 147–171 (2002).

[43] A. S. Barnard, M. Sternberg, Crystallinity and surface electrostatics of diamond nanocrystals. J. Mater. Chem. **17**, 4811–4819 (2007).

[44] J. Park , 3D structure of individual nanocrystals in solution by electron microscopy. Science **349**, 290–295 (2015). 26185247 10.1126/science.aab1343

[45] B. H. Kim , Critical differences in 3D atomic structure of individual ligand-protected nanocrystals in solution. Science **368**, 60–67 (2020). 32241943 10.1126/science.aax3233

[46] Q. Zhang , Swap motion–directed twinning of nanocrystals. Sci. Adv. **8**, eabp9970 (2022). 36206337 10.1126/sciadv.abp9970 PMC9544326

[47] H. Van Swygenhoven, Grain boundaries and dislocations. Science **296**, 66–67 (2002). 11935012 10.1126/science.1071040

[48] K.-N. Tu, A. Howie, Forbidden 200 diffraction spots in silicon. Philos. Mag. B **37**, 73–81 (1978).

[49] A. Ourmazd, G. R. Anstis, P. B. Hirsch, Dark-field electron microscopy of dissociated dislocations and surface steps in silicon using forbidden reflections. Philos. Mag. A **48**, 139–153 (1983).

[50] A. Ajoy , Orientation-independent room temperature optical 13C hyperpolarization in powdered diamond. Sci. Adv. **4**, eaar5492 (2018). 29795783 10.1126/sciadv.aar5492 PMC5959305

[51] Z. Shi , Deep elastic strain engineering of bandgap through machine learning. Proc. Natl. Acad. Sci. U.S.A. **116**, 4117–4122 (2019). 30770444 10.1073/pnas.1818555116 PMC6410806

[52] S. Plimpton, Fast parallel algorithms for short-range molecular dynamics. J. Comput. Phys. **117**, 1–19 (1995).

[53] A. M. Kamat, A. C. T. Van Duin, A. Yakovlev, Molecular dynamics simulations of laser-induced incandescence of soot using an extended ReaxFF reactive force field. J. Phys. Chem. A **114**, 12561–12572 (2010). 21067165 10.1021/jp1080302

[54] A. Stukowski, Visualization and analysis of atomistic simulation data with OVITO–the Open Visualization Tool. Model. Simul. Mater. Sci. Eng. **18**, 15012 (2009).

