# Supplementary material for: Unveiling the complexity of nanodiamond structures
Source: Proc Natl Acad Sci U S A. 2023 May 30;120(23):e2301981120. doi: 10.1073/pnas.2301981120 (PMC10266025; doi:10.1073/pnas.2301981120)
Supplement: Supplementary file 1 — Appendix 01 (PDF) [file pnas.2301981120.sapp.pdf]

Supporting Information

Unveiling the complexity of nanodiamond structures

Qi Zheng[1,2,3], Xian Shi[4,#], Jinyang Jiang[2,3], Haiyan Mao[5], Nicholas Montes[4], Nikolaos Kateris[4], Jeffrey A. Reimer[1,5], Hai Wang[4], Haimei Zheng[1,6,]*

[1] *Materials Sciences Division, Lawrence Berkeley National Laboratory, Berkeley, California 94720, United States*

[2] *School of Materials Science and Engineering, Southeast University, Nanjing 211189, P.R. China*

[3] *Jiangsu Key Laboratory for Construction Materials, Southeast University, Nanjing 211189, P. R. China.*

[4] *Department of Mechanical Engineering, Stanford University, Stanford, CA 94305, United States*

[5] *Department of Chemical and Biomolecular Engineering, University of California, Berkeley, California 94720, United States*

[6] *Department of Materials Science and Engineering, University of California, Berkeley, California 94720, United States*

[#] *Current address: Department of Mechanical and Aerospace Engineering, University of California, Irvine, California 92697, United States*

[*] *Correspondence to: hmzheng@lbl.gov*

This pdf includes these files:

Figure S1-16

Table S1

It covers the following contents: atomic structures of different diamonds; (200) forbidden spots observed in reported literature; the principles of double diffraction; reasoning in selecting the ReaxFF forcefield; defects captured in nanodiamonds using HRTEM; and some potential applications based on the (200) spots.

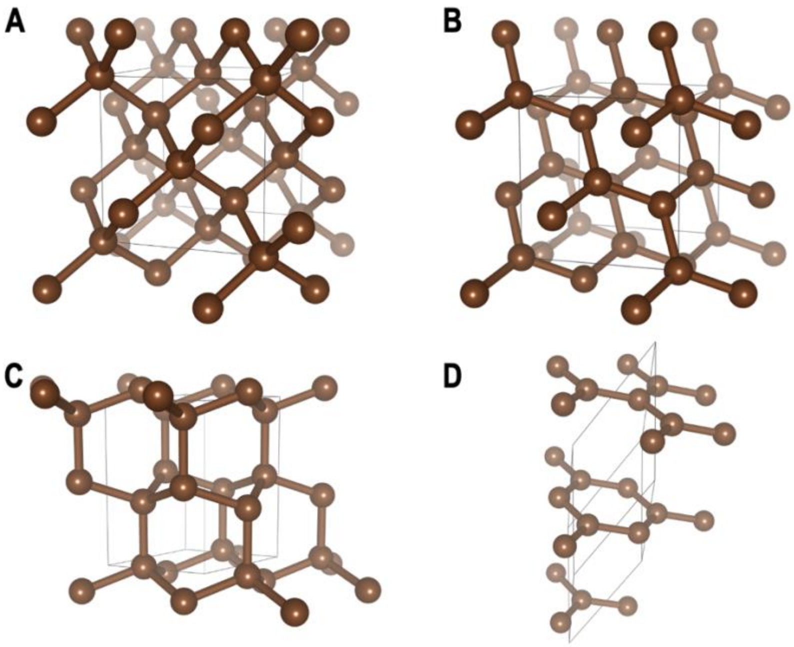

Figure S1. Crystal structures of diamond polymorphs, including (**A**) cubic diamond (1), (**B**) new diamond (2), (**C**) hexagonal diamond (3), and (**D**) i-carbon (4, 5). Note that the crystal files (.cif) can be found in the original references. To the best of our knowledge, only the cubic diamond structure has been resolved using X-ray and TEM techniques, while other structures are still under debate. Most structures of new diamond (6), hexagonal diamond, and i-carbon are proposed or optimized based on first-principles calculations and currently lack experimental validation.

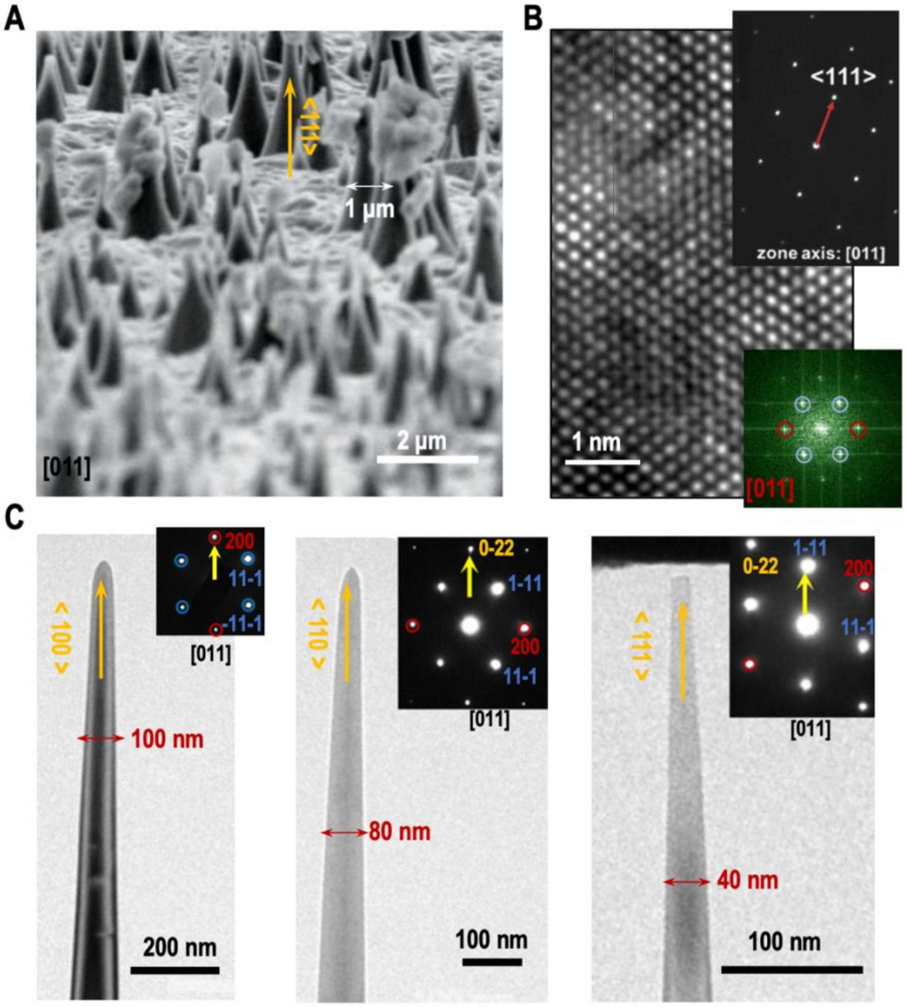

Figure S2. (200) forbidden reflections observed in cubic diamond particles due to double diffraction. (**A**) SEM micrograph of diamond nanoneedles and (**B**) its corresponding high-resolution TEM image. The diamond nanoneedle sample was synthesized through a plasma-enhanced chemical vapor deposition (CVD) method (7). Each nanoneedle is of ~1 μm size and grows along the <111> direction based on the electron diffraction pattern shown in the inset in (**B**). The (200) forbidden spots can be distinguished in the diffraction pattern and the FFT of the real-space TEM image. (**C**) Selected area electron diffraction (SAED) patterns of cubic diamond nanoneedles. Note that these needles were prepared and fabricated using the focused ion beam (FIB) method (8). The width/thickness of the nanoneedles all exceeds 40 nm, which can significantly induce double diffraction. In each corresponding electron diffraction pattern, the (200) spots can be captured with high intensity, marked in red. We found that the existence of (200) spots in the [011] zone axis can be well-explained using the "double diffraction" theory considering the large thickness of the sample, over 40 nm, in these references.

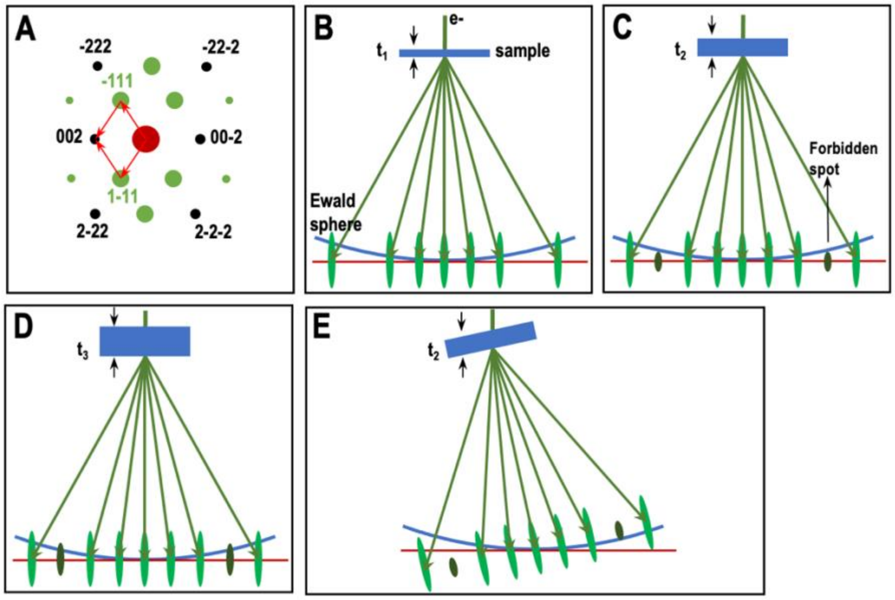

Figure S3. Illustrations on the exitance of the (200) forbidden spot and the principles of double diffraction. (**A**) The diffraction pattern of a cubic diamond crystal in the [110] zone axis. The reflections marked in black are extinct in the single scattering approximation when the crystal is very thin, while the ones marked in green exist in the patterns of both thin and thick crystals. When the crystal becomes thicker, multiple scattering occurs and thus these reflections can gain intensity and become visible because of more successive scattering events (even though they are probably weak if the sample is still relatively thin). For instance, electrons are indirectly scattered into the (002) reflection because of multiple scattering through the (1-11) and (-111) scattering vectors. The red arrows represent the multiple scattering paths for forming the visible (002) reflection. (**B**-**D**) Schematic illustrations of the reflection and forbidden spots of zone axis diffraction patterns at different specimen thicknesses. At zero tilt, the Ewald sphere is tangential to the zero-order Laue zone (ZOLZ) of the reciprocal lattices. (**B**) For very thin specimens, the forbidden spots (marked in dark green at a smaller size) do not appear, (**C**, **D**) while they appear when the thickness of the specimen increases, and the visible forbidden spot elongates more for thicker specimens (**D**). Note that the forbidden spots become more visible due to double diffraction in thicker specimens. (**E**) When the specimen is tilted, the locus of the intersection of the Ewald sphere with the spot does not change, and thus the projected positions of the reflections do not move. However, the excitation errors for the spots change significantly, so that reflection intensities in the electron diffraction patterns change significantly. Especially, for the relatively thin specimen as shown in (**E**), the forbidden spots will disappear on the recorded diffraction pattern (intersections of Ewald sphere with the spots) because they are too short to appear with specimen tilting. Tilting can be an examining method to check the double diffraction induced forbidden spots. Here, $t_1 < t_2 < t_3$. Higher Order Laue Zone (HOLZ) can also produce kinematically forbidden (002) reflections in F-43m and Fd-3m space groups.

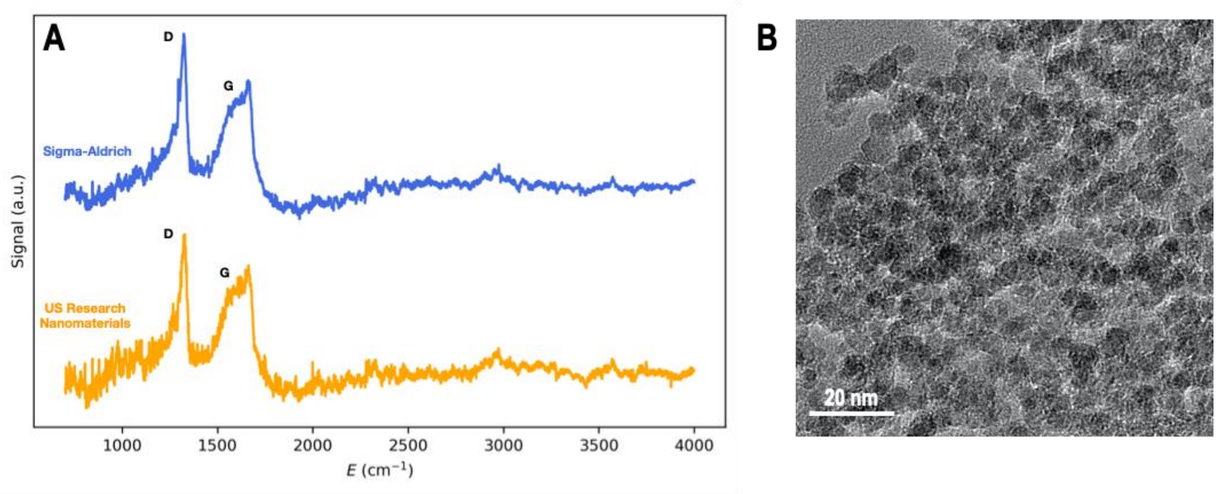

Figure S4. (**A**) Raman spectra of nanodiamond samples from Sigma-Aldrich (sample used in the main text) and from US Research Nanomaterials. Raman spectra were obtained using a Horiba Labram Raman Spectrometer with a 405 nm excitation laser (1.04 mW) and a 600 gr/mm grating. The Raman peaks were isolated from a broad photoluminescent signal by fitting a cubic polynomial to the data and subtracting it. The D peak located at 1323 cm^{-1} originates from the "diamond line" (triply degenerate T_{2g} mode) located at 1330 cm^{-1} in bulk diamond. Asymmetric broadening and slight red shifting are expected in nanodiamond samples due to phonon confinement (9). The broad G band located between 1450 and 1750 cm^{-1} is likely to comprise of several peaks associated with sp^2 stretching modes. The absence of a 2D band or a D' indicate the absence of graphitic carbon in the sample. We also characterized the diffraction patterns in detonation c-diamonds (US Research Nanomaterials). (**B**) TEM image of the detonation diamond nanoparticles (US Research Nanomaterials). The average size is ~ 5 nm.

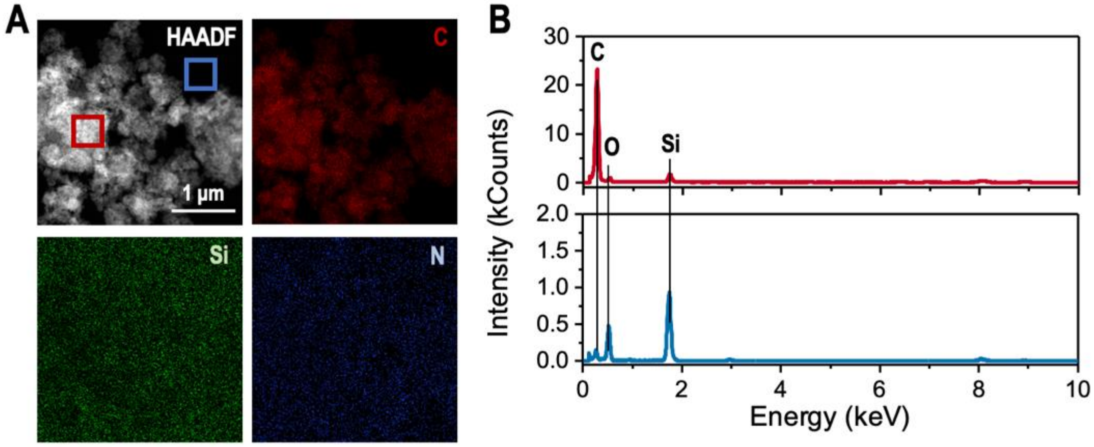

Figure S5. Elemental analysis of the nanodiamond samples. (**A**) EDS mapping of a nanoparticle domain and (**B**) the corresponding spectra. We found that the nanodiamonds are composed of C only, without noticeable impurities. From the EDS spectrum in (**B**), we can clearly conclude that the C signal is from the diamond domain (red square in the HAADF image) as only a very weak C signal is observed in an empty reference domain on the SiN_x substrate (blue square).

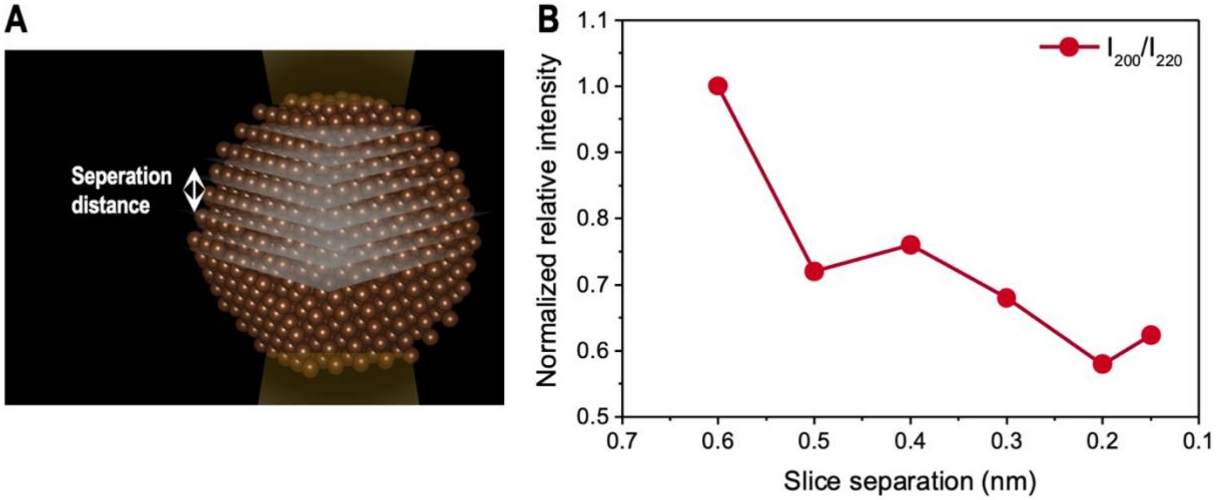

Figure S6. (**A**) An illustration on the separation distance. (**B**) The relation between normalized relative intensity of the (200) spot, and slice separation distance. The 3.7 nm cubic diamond nanoparticle was used as an example in our multislice simulations. The relative intensity, I_{200}/I_{220}, was examined. It can be observed that the normalized relative intensity fluctuated around 0.6 after the separation distance was narrowed to 0.3 nm. Note that the results do not vary significantly among different slice separation distances. As such, a 0.2 nm slice separation was employed throughout our simulations.

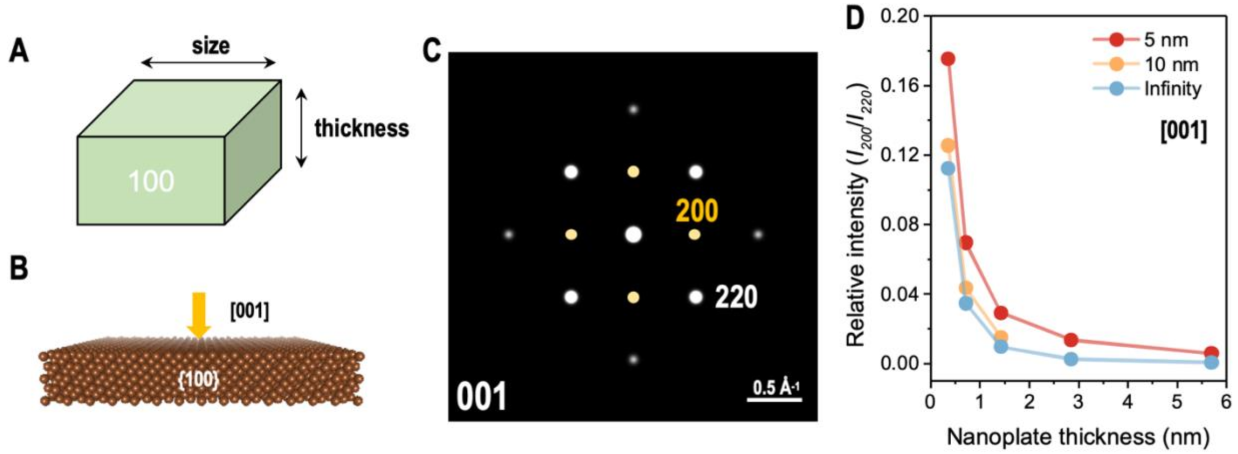

Figure S7. The relative intensity of the (200) spot in cubic diamond. Apart from the nanodiamond in the shape of an octahedron, cuboid diamond nanoparticles were also investigated at different sizes and thicknesses. (**A**) The nanodiamond cube was terminated with {100} facets, (**B**) and the diffraction pattern was simulated along the [001] zone axis. (**C**) The (200) forbidden spot can be observed in a thin nanoplate at ~1 nm thickness. (**D**) The relation between the relative intensity, I_{200}/I_{220}, and the nanoplate thickness.

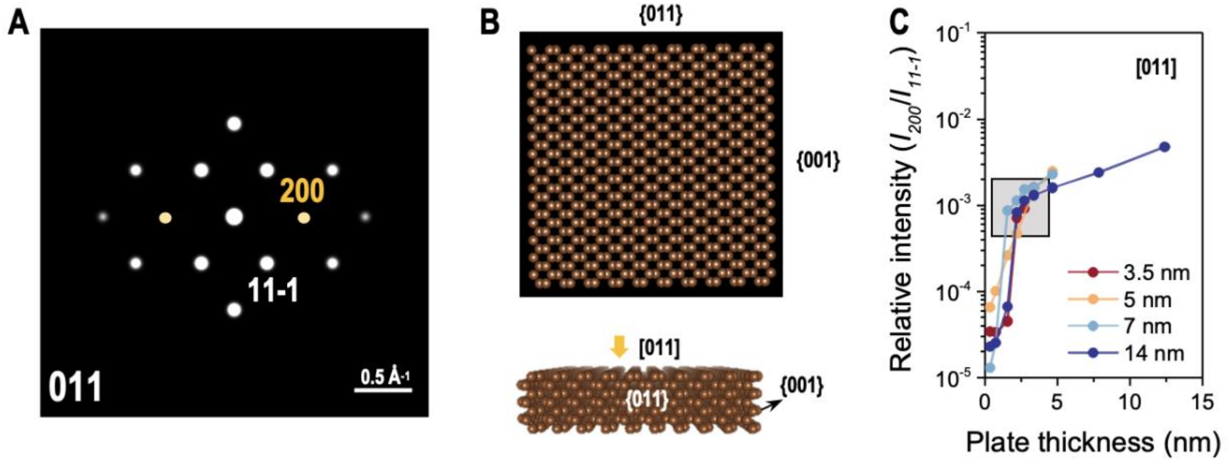

Figure S8. Benchmark case of double diffraction in nanosheets of different thicknesses. (**A**) Simulated electron diffraction pattern from the [011] zone axis. (200) spots are the forbidden reflections, which can be induced by the double diffraction effect. (**B**) Atomistic models of a cubic diamond nanoplate with its surface facets annotated. The {011} and {001} facets were terminated by the dangling C atoms, and the incident electron beam was passed through the [011] direction. (**C**) Relative intensity, $I_{200}/I_{11\text{-}1}$, as a function of nanoplate thickness. The relative intensity of (200) is higher in thicker nanoplates, which is consistent with the theory of double diffraction. The size in legend represents the planar size of the nanoplate, i.e., the side length of the square in (**B**). Also, the nanoplate at ~4 nm shares a similar relative intensity magnitude, at 10^{-3}, with the nanodiamond particles at a similar size. The benchmark study indicates the capability and consistency of our multi-slice simulation method, which includes the double diffraction effect. The method we used can be regarded as a first-principles simulation method considering all parameters.

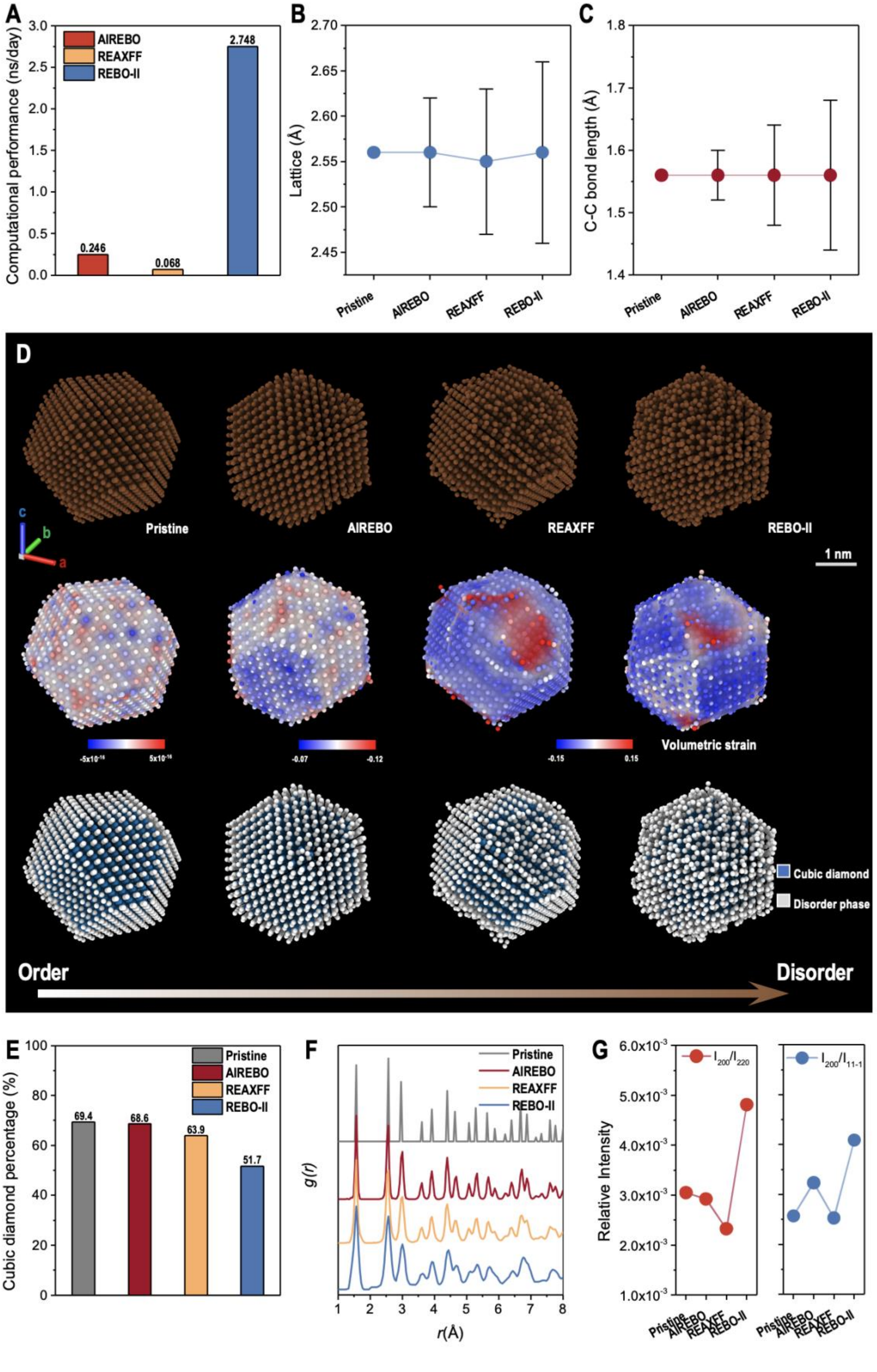
A
Computational performance (ns/day)
AIREBO
REAXFF
REBO-II
0.246
0.068
2.748
B
Lattice (Å)
Pristine
AIREBO
REAXFF
REBO-II
C
C-C bond length (Å)
D
Pristine
AIREBO
REAXFF
REBO-II
1 nm
Volumetric strain
Cubic diamond
Disorder phase
Order
Disorder
E
Cubic diamond percentage (%)
69.4
68.6
63.9
51.7
F
g(r)
r(Å)
G
Relative Intensity
I200/I220
I200/I11-1

Figure S9. Molecular dynamics simulations on the cubic diamond nanoparticles. Different forcefields were investigated including AIREBO (10), ReaxFF (11), and REBO-II (12). (**A**) Computational performance of simulations using different forcefields. REBO-II and AIREBO result in significantly lower cost, because of their shorter bond order-distance relation that is calculated within a cutoff distance, in contrast with the longer-range relation in ReaxFF. A 5 ns microcanonical ensemble (NVE) was employed to fully relax and optimize the diamond nanoparticles. (**B**) The optimized lattice spacing using different forcefields. We found that the optimal lattice resides at 2.56 Å and is independent of the forcefield used. (**C**) A similar trend can be observed in the optimized C-C bond length. The C-C bond is at ~1.55 Å. (**D**) The optimized configurations of nanodiamonds using different forcefields. Surface distortions were captured, particularly high strains presented at the corners and terraces. We noticed that the diamond nanoparticle simulated using the REBO-II forcefield exhibits the highest disorder while the AIREBO case is the most crystalline. Concerning the degree of disorder, we selected the ReaxFF forcefield as a balanced choice between the ordered and disordered phases. (**E**) The crystalline degree was further quantified using the cubic diamond fraction. The percentage was calculated based on the atom coordination, which is elaborated in **Figure S13**. (**F**) The radial distribution function (RDF) was analyzed. The smoothness of the curves reveals the disorder features of the nanodiamonds after a structural relaxation using different forcefields; namely, the sharper the curve, the more crystalline. (**G**) The relative intensity of the (200) spots was examined using the diamond nanostructures at the end of each MD simulation. All the relative intensity values are of the same magnitude. Note that the nanodiamonds above are ~3.7 nm roughly in diameter.

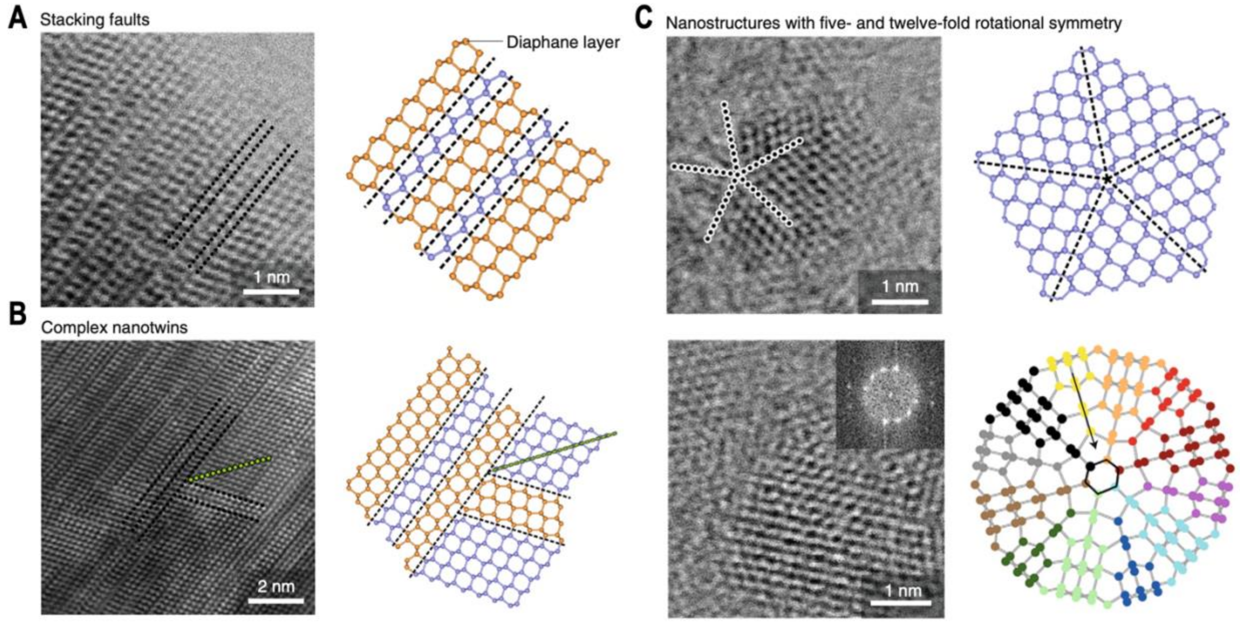

Figure S10. Structural complexity in diamond. Experimental evidence on the defects in nanodiamonds. (**A**) Cubic-hexagonal *sp*^{3}-bonded stacking faults and (**B**) complex patterns of nanotwins. HRTEM images of nanostructures revealing five- and twelve-fold rotational symmetries formed by multiple twinning and radially symmetric Mackay packing in the left column, with schematics in the right column. Figure adapted from: **A**, **B**, ref. (13); **C**, ref. (14).

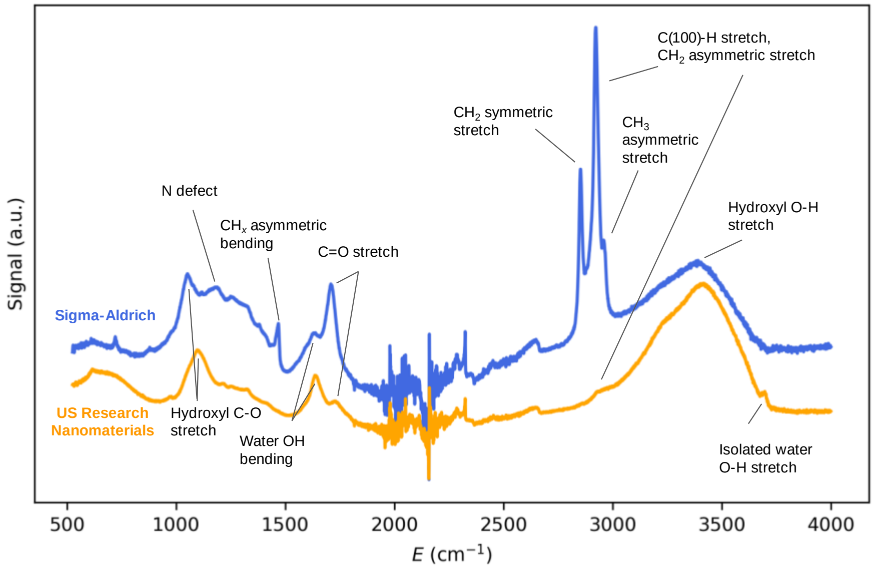

Figure S11. ATR-FTIR spectra of nanodiamond samples from Sigma-Aldrich and US Research Nanomaterials. FTIR spectra were obtained using a Nicolet iS50 FTIR Spectrometer operating in ATR mode with a diamond reflection cell. There is a significant difference in the surface termination between the two samples. Nanodiamonds from Sigma-Aldrich exhibit significant hydrogenation of the surface. Both samples also exhibit hydroxyl groups. Additionally, the presence of C=O stretch could be due to carboxyl groups on the surface. Note that the sample characterized in the main text is from Sigma-Aldrich with hydrogenation features. The US Research Nanomaterials diamond sample was used for double-checking the forbidden reflections in diffraction patterns with some results shown in **Figure S4**.

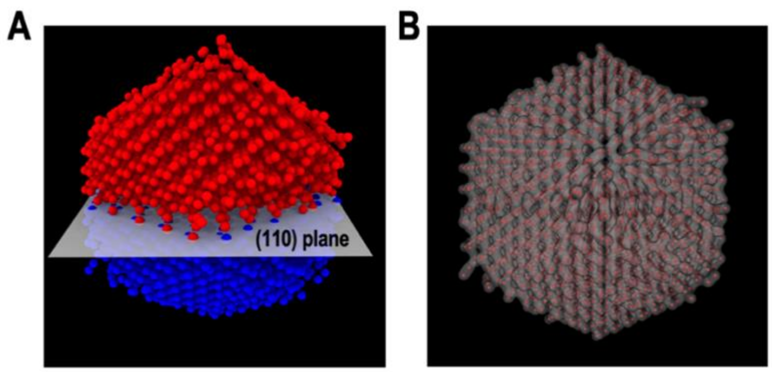

Figure S12. An artificial nanodiamond model with dislocations in the (110) plane. (**A**) The top (in red) and bottom (in blue) parts are slightly offset along the (110) plane within the structure. (**B**) Isosurfaces of the atoms in the diamond nanoparticle. The disorder features are apparent at the interface. Note that the nanodiamond is ~3.7 nm in diameter.

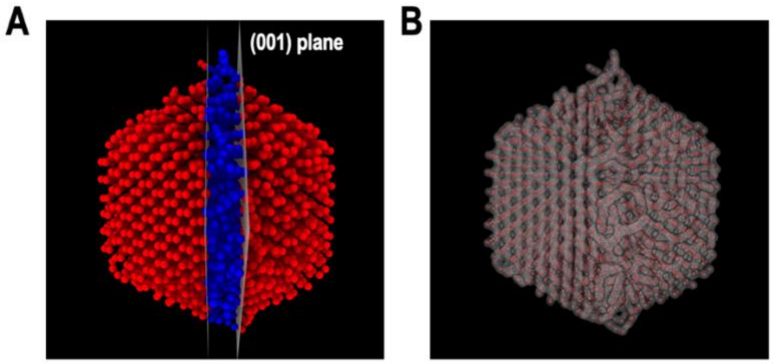

Figure S13. An artificial nanodiamond model with a grain boundary in the (001) plane. (**A**) The orientation of the left and right domains is different along the (001) plane, (**B**) causing the grain interface in the middle to be highly strained. Note that the nanodiamond is ~3.7 nm in diameter.

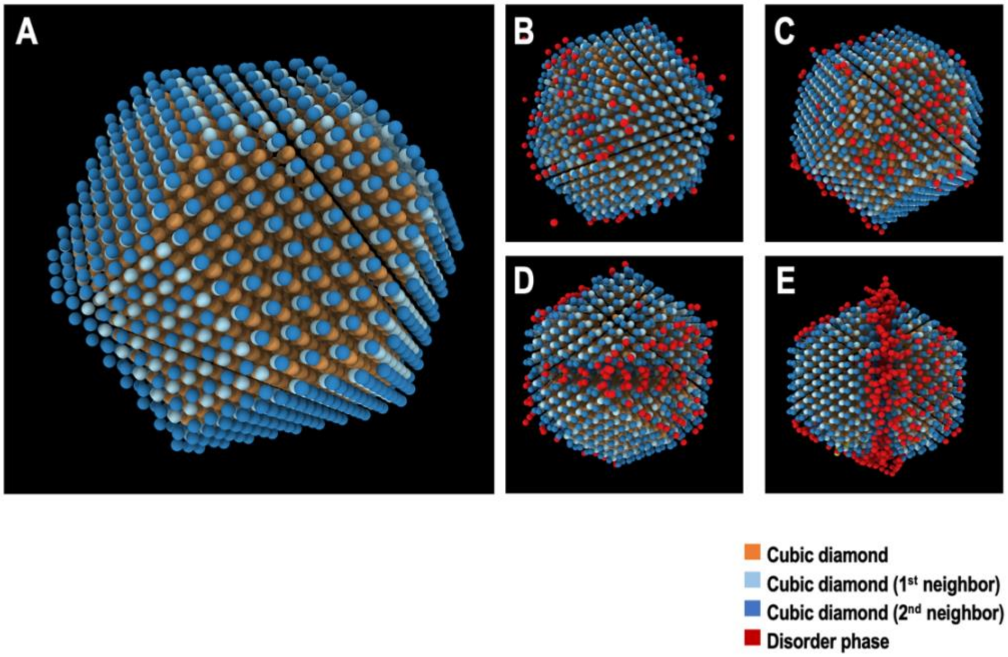

Figure S14. Identification of diamond structures, including (**A**) a perfect diamond lattice structure, (**B**, **C**) nanodiamonds with surface relaxations, and (**D**, **E**) nanodiamonds with different defects, such as dislocations and grain boundaries inside the particle. The algorithm analyzes the local environment of each atom up to the second neighbor shell to determine the local structural type (15). Different types and descriptions are listed in **Table S1**.

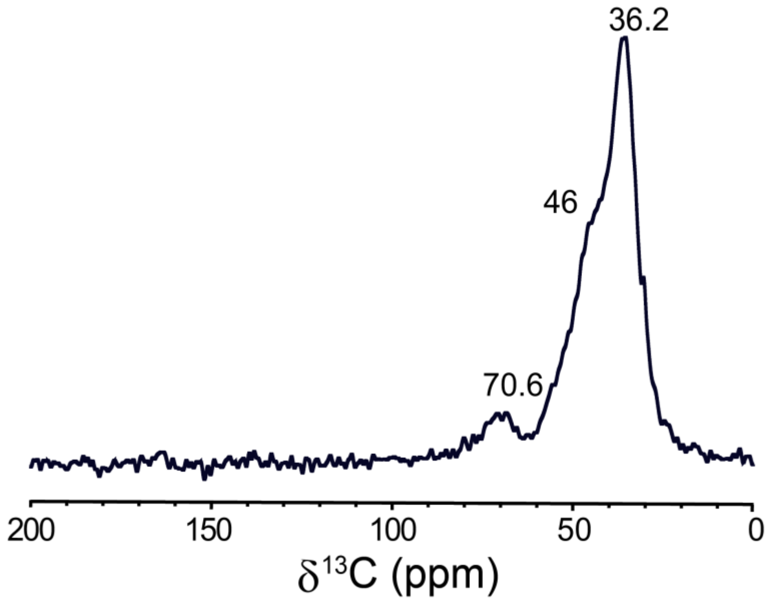

Figure S15. Solid-state ^{13}C NMR spectrum of nanodiamond particles. The solid-state ^{13}C cross-polarization magnetic angle spinning (CP-MAS) NMR spectrum was recorded at 16.4 T (700 MHz for 1H) using a Bruker 3.2 mm MAS probe at a spinning rate of 15 kHz with a radio frequency field strength of ~80 kHz.

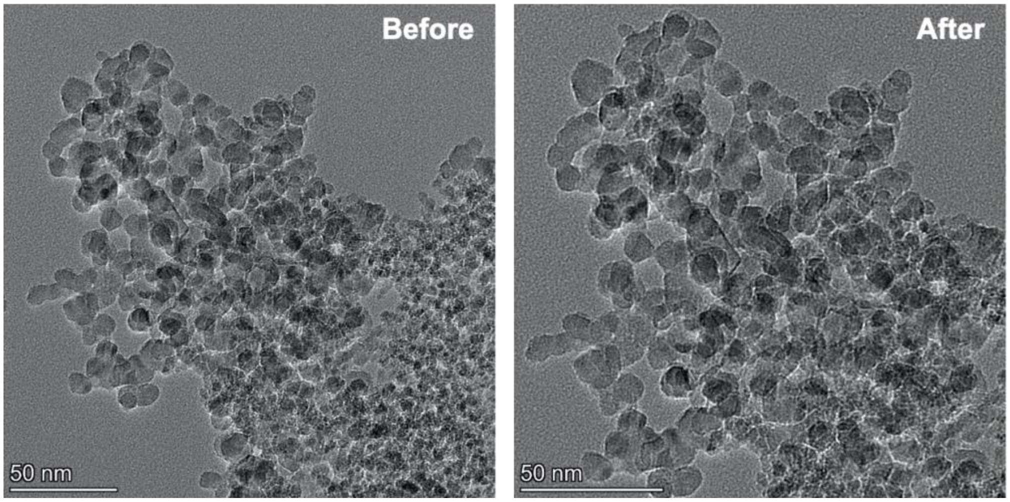

Figure S16. Evaluation of potential electron beam effects on nanodiamond characterization. We acquired the TEM images using a ThemIS transmission electron microscope with a Thermo Fisher Scientific Ceta CMOS camera, at an electron dose rate of 325 $e^{-}Å^{2}s^{-1}$, to avoid beam damage induced structural change. The results show that negligible changes can be found after the extended electron beam radiation with ~2 min. The image/diffraction acquisition time is 100-200 ms.

Table S1. The local environment of carbon atoms in diamond (15) in identification different diamond phases in **Figure S14**.

Type Name	Description
Cubic diamond	Atom having all its first and second nearest neighbors positioned on cubic diamond lattice sites.
Cubic diamond (1st neighbor)	Atom being a first neighbor of an atom that was classified as *cubic diamond*. Its four neighbors are positioned on lattice sites, but at least one of its second nearest neighbors is not.
Cubic diamond (2nd neighbor)	Atom being a second nearest neighbor of an atom that was classified as *cubic diamond*. The atom itself is positioned on a lattice site, but at least one of its neighbors is missing or is not positioned on a lattice site.
Other	Atom with unknown coordination structure, which doesn't belong to any of the classes below.

To classify a central atom, this structure identification method considers the second nearest neighbors to discriminate between cubic and hexagonal diamond structures. The method can be considered an extended version of the popular Common Neighbor Analysis (CNA), which is typically used to identify FCC, HCP, or BCC structures.

First, the nearest neighbors of an atom are identified. Then, for each of these four neighbors, their respective nearest neighbors are identified. This yields a list of the second nearest neighbors of the central atom. Finally, the CNA fingerprint is computed for these 12 second nearest neighbors and the central atom. If they are arranged in an FCC lattice, then the central atom is classified as the atom of the cubic diamond.

References

1. N. Bindzus, *et al.*, Experimental determination of core electron deformation in diamond. *Acta Crystallogr. Sect. A* **70**, 39–48 (2014).
2. B. Wen, J. Zhao, T. Li, C. Dong, N-diamond: An intermediate state between rhombohedral graphite and diamond? *New J. Phys.* **8**, 62 (2006).
3. F. P. Bundy, J. S. Kasper, Hexagonal diamond—a new form of carbon. *J. Chem. Phys.* **46**, 3437–3446 (1967).
4. H. Lipson, A. R. Stokes, A new structure of carbon. *Nature* **149**, 328 (1942).
5. M. J. Bucknum, C. J. PICKARD, I. Stamatin, E. A. Castro, On the structure of i-carbon. *J. Theor. Comput. Chem.* **5**, 175–185 (2006).
6. D. Li, *et al.*, Ab initio structure determination of n-diamond. *Sci. Rep.* **5**, 1–8 (2015).
7. A. Banerjee, *et al.*, Ultralarge elastic deformation of nanoscale diamond. *Science* **360**, 300–302 (2018).
8. A. Nie, *et al.*, Approaching diamond's theoretical elasticity and strength limits. *Nat. Commun.* **10**, 1–7 (2019).
9. M. Mermoux, S. Chang, H. A. Girard, J.-C. Arnault, Raman spectroscopy study of detonation nanodiamond. *Diam. Relat. Mater.* **87**, 248–260 (2018).
10. S. J. Stuart, A. B. Tutein, J. A. Harrison, A reactive potential for hydrocarbons with intermolecular interactions. *J. Chem. Phys.* **112**, 6472–6486 (2000).
11. A. M. Kamat, A. C. T. Van Duin, A. Yakovlev, Molecular dynamics simulations of laser-induced incandescence of soot using an extended ReaxFF reactive force field. *J. Phys. Chem. A* **114**, 12561–12572 (2010).
12. D. W. Brenner, *et al.*, A second-generation reactive empirical bond order (REBO) potential energy expression for hydrocarbons. *J. Phys. Condens. Matter* **14**, 783 (2002).
13. P. Németh, *et al.*, Lonsdaleite is faulted and twinned cubic diamond and does not exist as a discrete material. *Nat. Commun.* **5**, 1–5 (2014).
14. P. Németh, L. A. J. Garvie, P. R. Buseck, Twinning of cubic diamond explains reported nanodiamond polymorphs. *Sci. Rep.* **5**, 1–8 (2015).
15. E. Maras, O. Trushin, A. Stukowski, T. Ala-Nissila, H. Jónsson, Global transition path search for dislocation formation in Ge on Si(001). *Comput. Phys. Commun.* **205**, 13–21 (2016).
